# Supplementary material for: The Nero Lucano Pig Breed: Recovery and Variability
Source: Animals (Basel). 2021 May 7;11(5):1331. doi: 10.3390/ani11051331 (PMC8150585; doi:10.3390/ani11051331)
Supplement: Supplementary file 1 [file animals-11-01331-s001.zip › animals-1201319-supplementary.pdf]

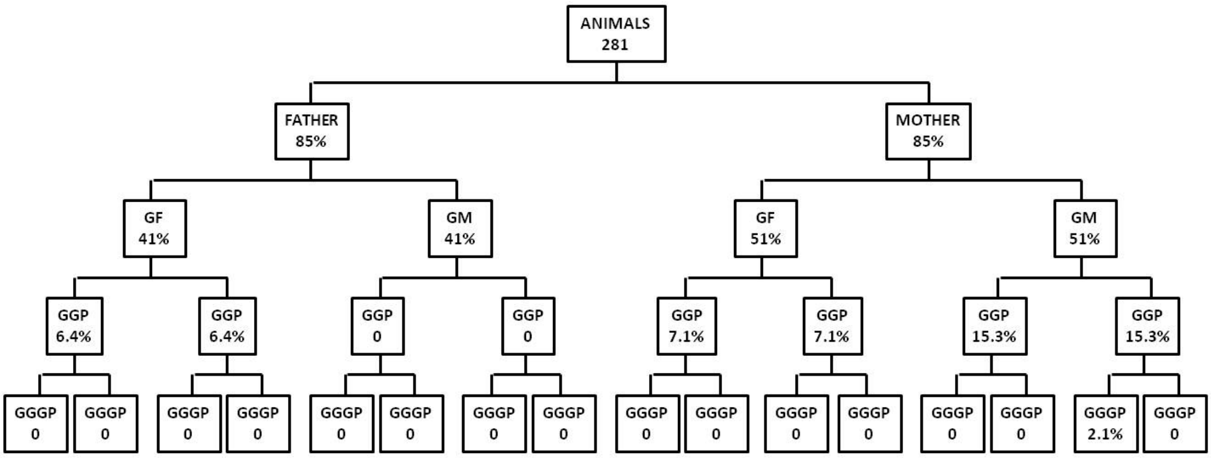

Figure S1. Pedigree completeness up to 4 generations back in Nero Lucano pig.

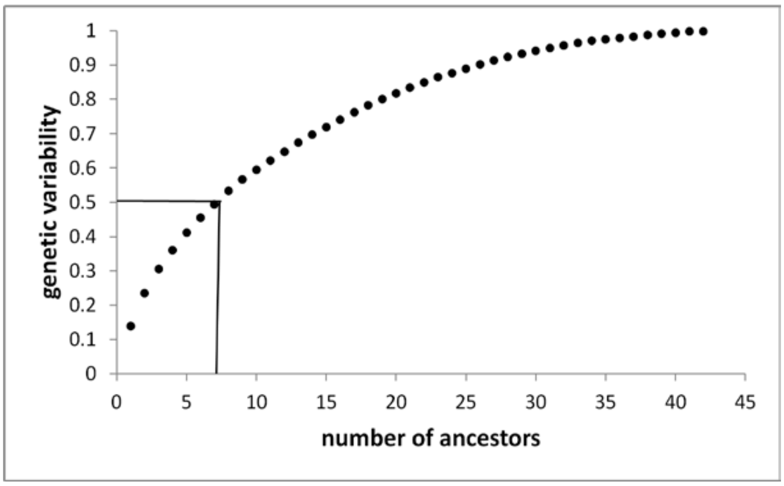

Figure S2. Effect of number of ancestors on genetic variability in Nero Lucano pig.

Table S1. distribution of SNPs per chromosome updated according to the Illumina PorcineSNP60 v2.0 Manifest File modified on 26/4/2019.

chromosome	previous SNP number	updated SNP number
0	7849	1091
1	6512	7183
2	3392	3767
3	2805	3263
4	3551	3902
5	2361	2740
6	3216	4122
7	3348	3610
8	2777	3178
9	3213	3528
10	1796	1954
11	1890	2151
12	1562	1803
13	4090	4604
14	3906	4267
15	2906	3303
16	1877	2086
17	1720	1916
18	1336	1487
X	1449	1426
Y	9	58
XY ψ-autosome		126
total	61565	61565

Table S2. Distribution of SNPs per chromosome and MAF classes in Nero Lucano pig.

SSC	MAF			total
	**0**	**0-0.05**	**0.05-0.5**	
0	207	286	503	996
1	1292	2011	3810	7113
2	339	1582	1791	3712
3	378	812	2027	3217
4	486	1927	1451	3864
5	340	1268	1102	2710
6	460	1006	2607	4073
7	487	1138	1919	3544
8	401	1132	1619	3152
9	366	1231	1882	3479
10	203	800	930	1933
11	204	667	1242	2113
12	153	835	788	1776
13	611	1944	2003	4558
14	576	1474	2163	4213
15	434	1307	1513	3254
16	183	511	1364	2058
17	196	744	944	1884
18	196	555	719	1470
23	405	328	669	1402
24	17	0	0	17
25	10	41	71	122
			**total**	60660

0= non-defined chromosome position

23=X chromosome

24=Y chromosome

25= XY [illegible]autosomal region

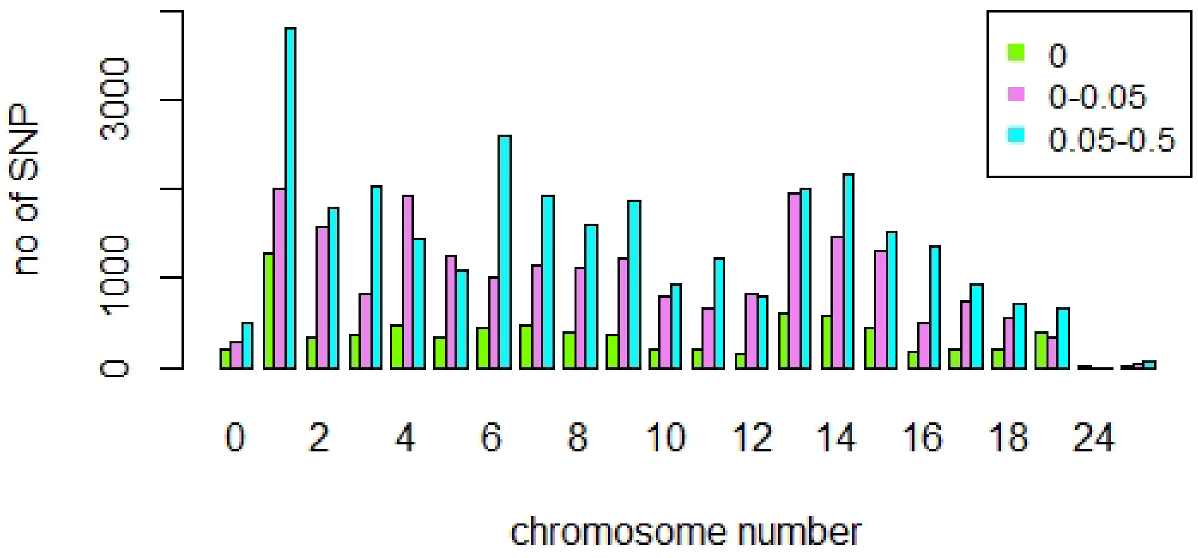

Figure S3. SNP distribution per chromosome and MAF classes in Nero Lucano pig. (0= non-defined chromosome position, 23=X chromosome, 24= Y chromosome, 25= XY ψ-autosomal region).

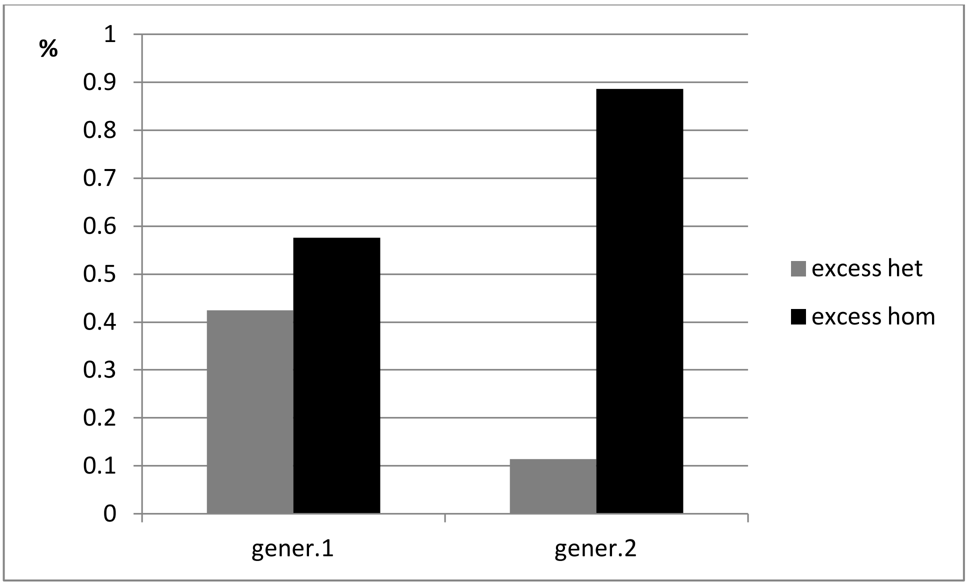

Figure S4. SNPs in Hardy-Weinberg disequilibrium due to excess of heterozygotes and homozygotes in generations 1 and 2 of the Nero Lucano pig pedigree.

Table S3. Distribution of the ROH per chromosome and ROH classes in Nero Lucano pig.

SSC	ROH class						% coverage
	<2Mb	2-4Mb	4-8Mb	8-16Mb	>16Mb	Totale N.	
1	136	517	171	154	280	1258	29.41
2	118	279	202	175	154	928	36.38
3	48	162	189	163	132	694	24.69
4	41	159	98	52	246	596	51.2
5	3	114	106	152	244	619	49.67
6	79	106	149	163	165	662	28.01
7	44	197	171	178	199	789	42.7
8	19	138	146	153	180	636	36.39
9	49	135	184	172	209	749	40.17
10	16	54	249	107	158	584	45.77
11	10	154	150	84	89	487	27.95
12	35	121	138	149	93	536	39.1
13	50	69	99	130	204	552	45.18
14	61	595	163	129	170	1118	41.44
15	13	88	167	118	222	608	48.86
16	39	106	95	88	91	419	29.28
17	53	95	134	85	120	487	40.48
18	6	74	174	82	101	437	39.18
N./class	820	3163	2785	2334	3057	12159	38.16
%/class	6.74	26.01	22.91	19.2	25.14	1	

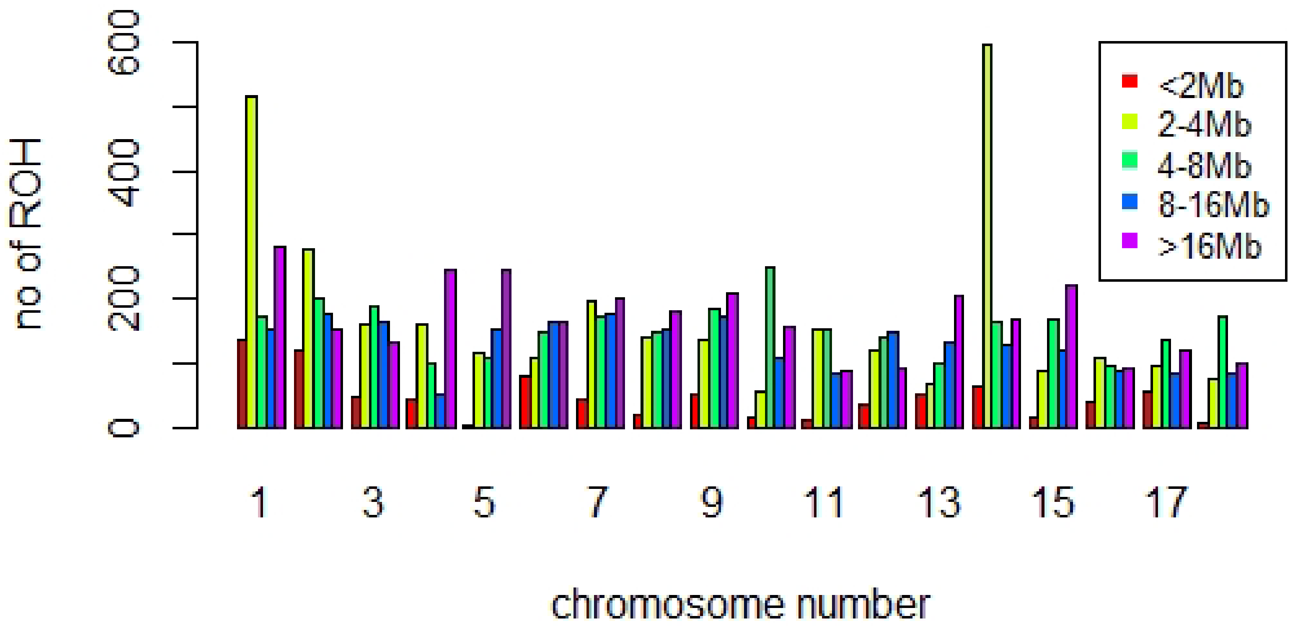

Figure S5. Distribution of the ROH per chromosome and length in Nero Lucano pig.

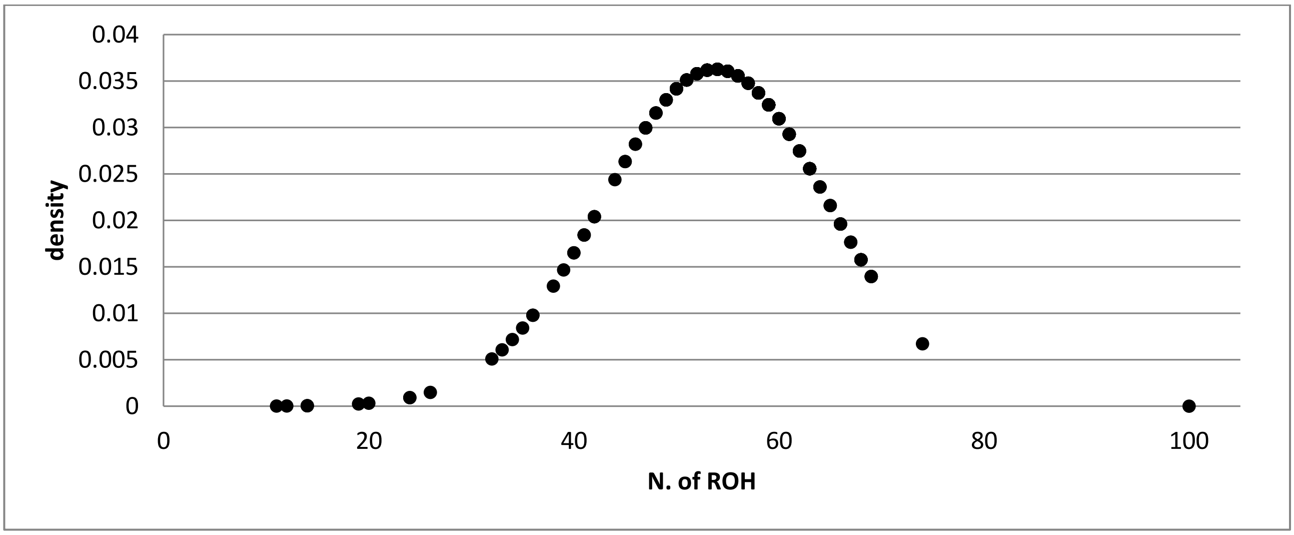

Figure S6. Distribution of the ROH in the Nero Lucano pig population.

Table S4. Distribution of the ROH present in more than 30% of the analyzed Nero Lucano pigs, longer than 500kb, and with at least 20 SNPs.

SSC	common ROH	the longer (kb)	the most represented (kb)
1	18	2908.297 (111)	756.477 (116)
2	17	2334.882 (75)	861.384 (195)
3	0	—	—
4	19	2676.525 (79)	1507.925 (205)
5	12	2790.301 (79)	2149.325 (210)
6	10	2901.328 (73)	1379.01 (105)
7	17	2810.644 (139)	1762.86 (149)
8	11	3894.132 (130)	773.528 (143)
9	10	1985.692 (155)	1290.151 (165)
10	6	1324.492 (99)	1059.501 (149)
11	3	1232.266 (110)	
12	4	1391.44 (72)	748.116 (125)
13	8	5555.072 (164)	2251.264 (166)
14	15	1872.548 (79)	1856.209 (214)
15	12	5749.139 (93)	2666.392 (201)
16	3	1030.413 (106)	
17	3	1033.309 (90)	765.889 (99)
18	3	1235.672 (101)	751.602 (197)

In parenthesis the number of pigs sharing the ROH

Table S5. Genes located in the most represented ROH per each chromosome of the Nero Lucano pig (NCBI Release 106, Chromosome Assembly Sscrofa 11.1). N=number of pigs sharing that ROH.

SSC	1	1	2	4	5	6	7	8	9	10	11	12	13	14	15	16	17	18
From bp	74,697,399	75,598,401	138,402,311	110,313,400	77,860,263	88,560,724	28,115,845	133,299,160	37,152,122	51,270,214	1,019,966	43,427,847	48,679,827	46,176,964	24,689,916	72,355,816	11,383,256	3,021,252
To bp	75,386,437	76,354,878	139,263,695	111,821,325	80,009,588	89,939,734	29,878,705	134,072,688	38,442,273	52,329,715	2,252,232	44,175,963	51,136,679	48,033,173	27,356,308	73,387,229	12,149,145	3,772,854
N	116	116	195	205	210	105	149	143	165	149	110	125	166	214	201	106	99	197
GENES	FOXO3	AK9	TRPC7	EPS8L3	RPAP3	KHDRBS1	PRIM2	ARHGAP24	DDX10	OTUD1	IFT88	RAB11FIP4	SUCLG2	ZNRF3	STEAP3	TAS2R1	POLB	AK9
	ARMC2	FIG4	SPOCK1	GSTM3	ENDOU	TMEM39B	RAB23	WDFY3	ZC3H12C	PTF1A	IL17D	NF1	FAM19A1	KREMEN1	DBI	SEMA5A	DKK4	FIG4
	SESN1	GPR6		AMPD2	RAPGEF3	KPNA6	BAG2	CDS1	RDX	MSRB2	XPO4	EVI2A	FAM19A4	EMID1	PROC		VDAC3	GPR6
	CEP57L1	WASF1		GNAT2	SLC48A1	TXLNA	ZNF451		FDX1	ARMC3	LATS2	EVI2B	EOGT	RHBDD3	MAP3K2		SLC20A2	WASF1
		CDC40		GNAI3	HDAC7	CCDC28B	BEND6			PIP4K2A	SAP18	OMG	TMF1	EWSR1	ERCC3		SMIM19	CDC40
		METTL24		AMIGO1	VDR	IQCC	DST				SKA3	WSB1	UBA3	GAS2L1	BIN1		CHRNB3	METTL24
				ATXN7L2	TMEM106C	DCDC2B	COL21A1				MRPL57	KSR1	ARL6IP5	RASL10A	TEX51		CHRNA6	
				SYPL2	COL2A1	TMEM234	VPS52				ZDHHC20	LGALS9	LMOD3	AP1B1	GYPC		THAP1	
				PSMA5	SENP1	EIF3I	RPS18				MICU2	NOS2	FRMD4B	NEFH	CNTNAP5		RNF170	
				SORT1	PFKM	FAM167B	B3GALT4				FGF9			THOC5			HOOK3	
				MYBPHL	ASB8	LCK	WDR46							NIPSNAP1			FNTA	
				PSRC1	CCDC184	HDAC1	PFDN6							NF2			POMK	
				CELSR2	ZNF641	MARCKSL1	RGL2							CABP7			HGSNAT	
				SARS	LALBA	FAM229A	TAPBP							ZMAT5			INTS10	
				KIAA1324	KANSL2	BSDC1	ZBTB22							UQCR10			CSGALNACT1	
				TMEM167B	APPL2	TSSK3	DAXX							ASCC2				
				TAF13	WASHC4	ZBTB8B	KIFC1							MTMR3				
				WDR47	ALDH1L2	ZBTB8A	PHF1							LIF				
				CLCC1	SLC41A2	ZBTB8OS	CUTA							OSM				
				GPSM2	CHST11	RBBP4	SYNGAP1							CASTOR1				
				AKNAD1		SYNC	ZBTB9							TBC1D10A				
				STXBP3		KIAA1522	BAK1							SF3A1				
				FNDC7		YARS	ITPR3							CCDC157				
				PRPF38B		S100PBP								RNF215				
				HENMT1		FNDC5								SEC14L2				
				FAM102B		HPCA								MTFP1				
				SLC25A24		TMEM54								GAL3ST1				
						RNF19B								PES1				
						AK2								TCN2				
						AZIN2								SLC35E4				
						TRIM62								DUSP18				
						ZNF362								OSBP2				
						A3GALT2								HSBP1P				
						PHC2								MORC2				
						ZSCAN20								SMTN				
						CSMD2								SELENOM				
														INPP5J				
														PLA2G3				
														RNF185				
														LIMK2				
